# Visual Contrast Enhancement Algorithm Based on Histogram Equalization

**DOI:** 10.3390/s150716981

**Published:** 2015-07-13

**Authors:** Chih-Chung Ting, Bing-Fei Wu, Meng-Liang Chung, Chung-Cheng Chiu, Ya-Ching Wu

**Affiliations:** 1 School of Defense Science, Chung Cheng Institute of Technology, National Defense University, Taoyuan 33551, Taiwan; E-Mail: chihchungting@gmail.com; 2 Institute of Electrical and Control Engineering, National Chiao Tung University, Hsinchu 30010, Taiwan; E-Mails: bwu@cssp.cn.nctu.edu.tw; 3 Department of Electrical and Electronic Engineering, Chung Cheng Institute of Technology, National Defense University, Taoyuan 33551, Taiwan; E-Mails: davidchiu@ndu.edu.tw (C-C.C.); m0919048774@yahoo.com.tw (Y-C.W.)

**Keywords:** contrast enhancement, dynamic range, histogram equalization (HE), just-noticeable difference (JND)

## Abstract

Image enhancement techniques primarily improve the contrast of an image to lend it a better appearance. One of the popular enhancement methods is histogram equalization (HE) because of its simplicity and effectiveness. However, it is rarely applied to consumer electronics products because it can cause excessive contrast enhancement and feature loss problems. These problems make the images processed by HE look unnatural and introduce unwanted artifacts in them. In this study, a visual contrast enhancement algorithm (VCEA) based on HE is proposed. VCEA considers the requirements of the human visual perception in order to address the drawbacks of HE. It effectively solves the excessive contrast enhancement problem by adjusting the spaces between two adjacent gray values of the HE histogram. In addition, VCEA reduces the effects of the feature loss problem by using the obtained spaces. Furthermore, VCEA enhances the detailed textures of an image to generate an enhanced image with better visual quality. Experimental results show that images obtained by applying VCEA have higher contrast and are more suited to human visual perception than those processed by HE and other HE-based methods.

## 1. Introduction

Light plays a crucial role in generating images of satisfactory quality in photography. Strong light causes an image to have a washed out appearance; on the contrary, weak light leads to an image that is too dark to be visible. In these two cases, the contrasts of the images are low and their detailed textures are difficult to discern. Furthermore, the poor sensitivity of charge-coupled device/complementary–metal–oxide–semiconductor (CCD/CMOS) sensors leads to images with excessively narrow dynamic ranges and renders their details unclear. Consequently, image enhancement techniques are widely used to solve such problems and improve image quality.

Histogram equalization (HE) [1] is a popular image contrast enhancement technique because of its simplicity and effectiveness. The image processed by HE usually has a higher contrast and better visual effects. Although HE can effectively enhance a low-contrast image, it can overstretch the distances between two neighboring gray values of the image and cause the excessive contrast enhancement problem. Furthermore, it can cause the feature loss problem by merging many gray values with small probabilities into a single gray value.

Many researchers have proposed methods to solve the above-mentioned drawbacks of HE. A few have attempted to solve the excessive contrast enhancement problem. Kim [2] proposed brightness preserving bi-histogram equalization (BBHE), which divides the histogram of an image into two parts, based on its mean, and equalizes them using HE. Abdullah-Al-Wadud *et al*. [3,4] proposed dynamic histogram equalization (DHE), which uses local minima to divide the histogram into several subhistograms. If a subhistogram is not normally distributed, DHE divides it into three parts according to the values of μ + σ and μ − σ, where μ and σ are the mean and standard deviation of the subhistogram, respectively. Each subhistogram is then assigned a new dynamic range, and HE is applied to each. Park *et al*. [5] proposed dynamic range separate histogram equalization (DRSHE), which uses the weighted average of absolute color difference (WAAD) to render the original image more uniformly distributed. DRSHE divides the dynamic range of the histogram into four equal subhistograms and resizes each grayscale range according to its area ratio. Following this, DRSHE uniformly redistributes the intensities of the histogram in the resized grayscale range. Lin *et al*. [6] proposed statistic-separate tri-histogram equalization (SSTHE), which divides the histogram of an image into three subhistograms based on the mean and standard deviation of the image. The span of each subhistogram is then stretched, and HE is applied to each. Ooi *et al*. [7] proposed bi-histogram equalization with a plateau level (BHEPL), which is an extension of BBHE. Like BBHE, BHEPL separates the input histogram into two subhistograms based on the mean of the relevant image. It then determines two plateau limits and accordingly clips the two subhistograms in order to avoid over-amplification of noise. Following this, the two subhistograms are separately equalized by utilizing two transform functions. Wu *et al*. [8] proposed weighting mean-separated sub-histogram equalization (WMSHE) method that divides a histogram of an image into six subhistograms according to the proposed weighting mean function, and performs HE within each subhistogram. All the above methods involve using different methods to segment the histogram into several subhistograms, and then using HE or other equalization methods to enhance the images. They are able to solve the excessive contrast enhancement problem because each subhistogram is restricted to a new range. However, they cannot solve the feature loss problem caused by HE or HE-based methods.

Furthermore, a growing number of studies have proposed methods to preserve the brightness of images and maintain image quality. Kim proposed BBHE [2] to maintain a mean value of the enhanced image that is close to that of the input image. Wongsritong *et al*. [9] proposed multi-peak histogram equalization with brightness preserving (MPHEBP), which uses the peaks of the histogram to divide it into several regions, and performs HE within each region. It can preserve the mean brightness of an input image. Wang *et al*. [10] proposed equal area dualistic sub-image histogram equalization (DSIHE), which divides an image into two equal area subimages based on its median value, and performs HE within each subimage. The contrast of an image enhanced by the DSIHE method is the average of the segmentation gray level and the middle-gray level of the gray scale of the image. Therefore, DSIHE preserves brightness. Chen *et al.* [11] proposed a method called recursive mean-separate histogram equalization (RMSHE), which is an extension of BBHE, to preserve the brightness of images. Like BBHE, RMSHE separates the given histogram into two subhistograms using its mean. It performs the division r times. The enhanced image generated by RMSHE can satisfactorily preserve brightness. Chen *et al*. proposed a minimum mean brightness error bi-histogram equalization (MMBEBHE) [12], which calculates all absolute mean brightness error (AMBE) values for intensity levels 0 to *L* − 1, and determines the threshold value that produces the minimum absolute difference between the input and output means. MMBEBHE then separates the entered histogram into two subhistograms based on the threshold value and equalizes them. It can provide maximum brightness preservation of the original image. Wang and Ye [13] proposed the brightness-preserving histogram equalization with the maximum entropy (BPHEME), which determines a specified histogram that preserves the mean brightness of the original image and has maximum entropy. Therefore, BPHEME can preserve image brightness. Like DSIHE, recursive sub-image histogram equalization (RSIHE) proposed by Sim *et al*. [14] uses the median value to recursively divide the image r times, and performs HE on each subimage. As in DSIHE, the average brightness of the processed image is the average of the segmentation gray level and the middle-gray level of the grayscale of the image. Thus, RSIHE can preserve brightness. Ibrahim *et al*. [15] proposed brightness-preserving dynamic histogram equalization (BPDHE), which is an extension of MPHEBP [9] and DHE [3,4]. Like MPHEBP, BPDHE segments a histogram based on the local maxima of the smoothed histogram. Before equalizing each segment, it maps it to a new dynamic range. This process is similar to that used in DHE. The average intensity of the resultant image of BPDHE is nearly the same as the one of the input image. Wang *et al*. [16] proposed flattest histogram specification with accurate brightness preservation (FHSABP), which tries to determine the optimal histogram, the flattest one with the mean brightness constraint. FHSABP then uses an exact histogram specification to obtain better brightness preservation. Ooi *et al*. [17] proposed dynamic quadrants histogram equalization plateau limit (DQHEPL), which divides a histogram based on its median and iteratively produces four subhistograms. DQHEPL then calculates each plateau limit, and clips each subhistogram by its plateau limit. Following this, each subhistogram is assigned a new dynamic range and HE is applied to each. The images processed by DQHEPL can maintain mean brightness. Thomas *et al*. [18] adopted the concepts of BPHEME [13] and piecewise linear transformation (PLT) [19] to propose a piecewise maximum entropy (PME) method. PME uses the piecewise transformation function to avoid a mean value too far from the original mean and maximizes entropy. The resulting image processed by PME preserves the original brightness quite well. All the above methods attempt to overcome the drawback of significant changes in brightness caused by HE by maintaining the brightness of the input image as far as possible in order to enhance it. They can generate images that retain almost the same brightness as that of the original. However, when the input image is underexposed or overexposed, maintaining its brightness is not reasonable because it is unsuitable for human visual perception.

Therefore, in this paper, a visual contrast enhancement algorithm (VCEA) considering the characteristics of human visual perception is proposed. This algorithm mitigates the excessive contrast enhancement and the feature loss problems of HE. Furthermore, VCEA enhances the detailed textures of an image. Images processed by VCEA have better visual quality and are better suited to human visual perception than those processed by HE and other HE-based methods.

This paper is organized as follows. The proposed VCEA algorithm is introduced in Section 2. Section 3 is devoted to experimental results to compare the performance of VCEA with HE and other HE-based methods. Finally, conclusions are provided in Section 4.

## 2. Visual Contrast Enhancement Algorithm (VCEA)

Histogram equalization (HE) is a well-known technique to enhance the contrast of images because of its simplicity and effectiveness. However, HE is rarely applied directly to consumer electronics products because it can cause the excessive contrast enhancement and feature loss problems. Although many research studies have proposed methods to overcome the excessive contrast enhancement problem in HE, they have not considered the problem of the compression of gray values, which results in the loss of a few features in the enhanced image. Starting from the strategy adopted by past studies in the area, a visual contrast enhancement algorithm (VCEA) based on HE is proposed. It considers the requirements of the human visual perception in order to solve the excessive contrast enhancement problem and the feature loss problem caused by HE. Furthermore, VCEA enhances the detailed textures of images and improves the quality of enhanced images.

**Figure 1:**
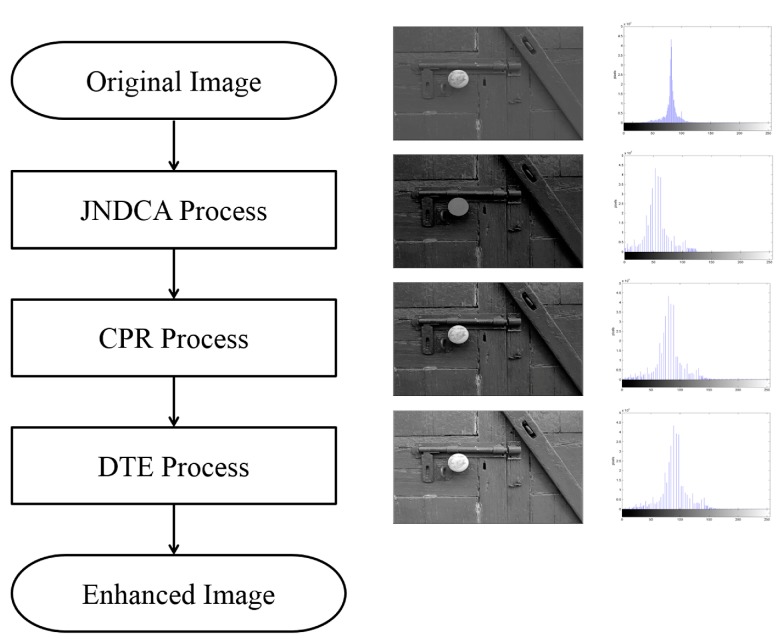
Functional block diagram of visual contrast enhancement algorithm (VCEA).

VCEA has three major processes: just-noticeable difference contrast adjustment (JNDCA), compressed pixel recovery (CPR), and detailed texture enhancement (DTE). The functional block diagram of VCEA is shown in Figure 1. The details of the three processes of VCEA are as follows:

### 2.1. Just-Noticeable Difference Contrast Adjustment (JNDCA)

The main purpose of the JNDCA process is to address the excessive enhancement problem of overstretched space between two adjacent gray values caused by HE and enable the enhanced image to satisfy the requirements of human visual perception. Before introducing the JNDCA process, just-noticeable difference (JND) needs to be clarified.

JND is a quantitative measure used to distinguish the luminance change perceived by the human visual system. It is defined as the amount of light $∆B_{T}$ necessary to add to a visual field of intensity *B* such that it can be discriminated from the background [20,21]. It has been widely used in different applications, such as watermarking, image enhancement, data hiding, *etc*., in recent years. Lie and Chang [21] proposed the least-significant bit (LSB) mapping function, which provides the number of LSBs embedded for each gray value according to the sensitivity of human visual perception to changes in image contrast. In this paper, a space adjustment function *S*(*x*) is referred to the function proposed by Lie and Chang and devised, which shortens the spaces between two adjacent gray values of the HE histogram to satisfy the minimum discernment requirements of human visual perception for the contrast change in each gray value. The space adjustment function is as follows:

(1) $$S(x)=\left\{ \begin{array}{lll} 2 & 0\leq x\leq 85 & \\ 3 & 86\leq x\leq 182 & where\;x\;is\;the\;gray\;value\\ 4 & 183\leq x\leq 255 & \end{array}\right.$$

The JNDCA process uses the space adjustment function to adjust the spaces between adjacent gray values in order to improve the excessive contrast enhancement problem of HE. It is assumed that *HEhist*(*x*) is the total number of pixels of the HE histogram at gray value *x*, where *x* ranges from 0 to 255, and the space between gray value *x* − 1 and *x* is *l*. When *l* is greater than the space adjustment function *S*(*x*), *HEhist*(*x*) is shifted back by *l* − *S*(*x*) gray levels. On the contrary, when *l* is equal to or less than *S*(*x*), it implies that no space to be adjusted; thus, *HEhist*(*x*) remains at its original location. Once all *HEhist*(*x*) have sequentially been shifted back, the JNDCA image and available spaces are obtained.

**Figure 2:**
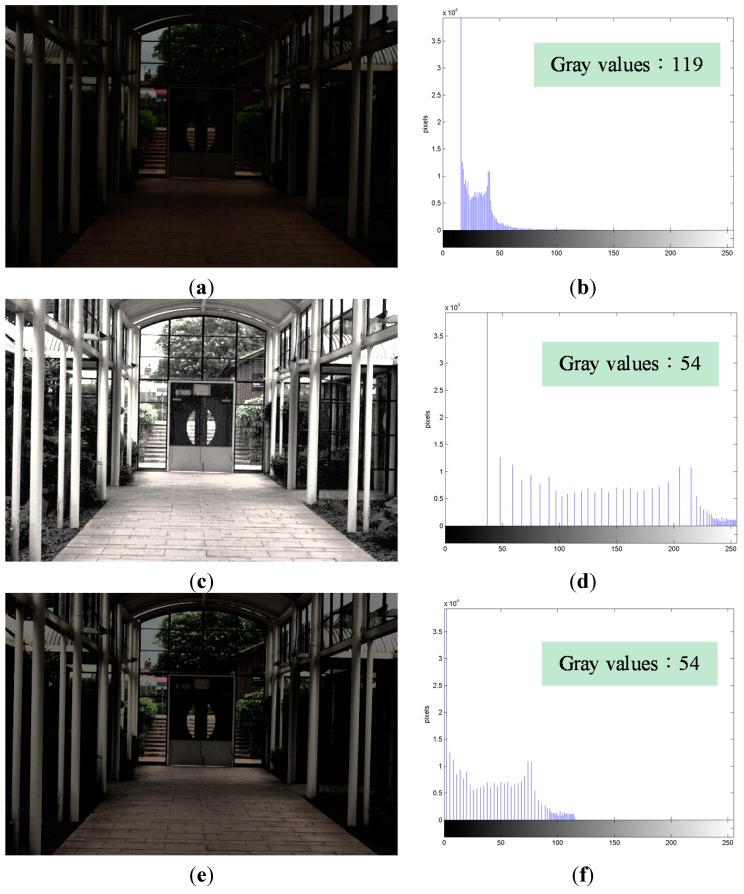
“Indoor View” [22] (image size: 640 × 428 pixels) processed by just-noticeable difference contrast adjustment (JNDCA). (**a**) Original image; (**b**) Original histogram; (**c**) Histogram Equalization (HE) image; (**d**) HE histogram; (**e**) JNDCA image; (**f**) JNDCA histogram.

Figure 2a shows an underexposed image containing 119 gray values. The image in Figure 2c is processed by HE and suffers from an excessive enhancement problem: the door, floor, rain shelter, *etc*., are over-enhanced. Moreover, this image has only 54 gray values because multiple gray values are compressed because of HE. As a result, Figure 2c suffers from the feature loss problem, which causes the textures of the rain shelter to disappear. Figure 2e shows the JNDCA image obtained by applying the JNDCA process. It contains 54 gray values, which is the same as that in Figure 2c. Thus, this image satisfies the minimum discrimination requirement of human visual perception. Through space adjustment between two neighboring gray values, Figure 2e shows improvement in the excessive contrast enhancement problem caused by HE. Figure 2b,d, and f are the histograms of the luminance (Y) component of Figure 2a,c, and e, respectively. The obtained available spaces, “free spaces,” are used in the following processes for further enhancement of image quality.

### 2.2. Compressed Pixel Recovery (CPR)

The CPR process mainly addresses the feature loss problem caused by HE or HE-based methods. The principle underlying HE is the enhancement of the contrast of an image by stretching its dynamic range from gray level 0 to 255 based on the cumulative distribution function (CDF). When the cumulative probability of a certain gray value is less than 1/255, the gray value is not allocated a gray level space and is merged into other gray values. In this condition, many gray values are merged into a specific gray value, which leads to the feature loss problem. To address the problem, the CPR process uses free spaces to recover as many compressed gray values as possible in order to regain the lost features in the enhanced image.

The CPR process is as follows. It is assumed that *JNDCAhist*(*x*), *CPRhist*(*x*), and *histogram*(*x*) are the total number of pixels in the JNDCA, CPR, and the original histograms at gray value *x*, respectively, where *x* ranges from 0 to 255. The CPR process first compares the JNDCA histogram with the original histogram. When *JNDCAhist*(*x*) is not zero, the CPR process determines the range of gray levels of the original histogram containing the sum of pixels, which is equal to *JNDCAhist*(*x*)*.* Following this, the CPR process recovers the pixels in the particular range of gray levels from the original histogram, and repeats the same task until all the free spaces are used up. For example, when the value of *JNDCAhist*(*x*) at gray value *x* is equal to the cumulative pixels from gray level *y* to *z* of the original histogram,

(2) $$JNDCAhist(x)=\sum_{X_{i}=y}^{z}histogram(X_{i})$$

Having recovered the lost features, gray value *x* assumes the range x,  x + 1, …, x + (z − y) in the CPR image. The pixels of each recovered gray value *x* can be expressed as:

(3) $$CPRhist(x_{i})=histogram(y+x_{i}-x)\;x_{i}=x,x+1,.....,x+(z-y)$$

**Figure 3:**
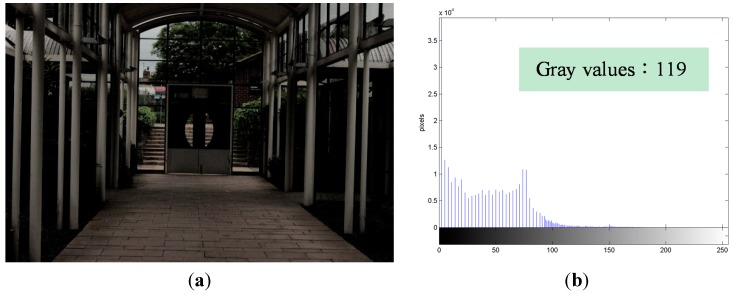
“Indoor View” processed by compressed pixel recovery (CPR). (**a**) CPR image; (**b**) CPR histogram.

Figure 3a is obtained by applying the CPR process. Through this process, many compressed gray values such as the textures of the rain shelter are recovered. Figure 3a contains 119 gray values, which is the same number as that in the original image. The CPR process effectively mitigates the feature loss problem caused by HE. It also makes Figure 3a appear better than the JNDCA image because the lost features are recovered in the CPR image. Figure 3b is the histogram of the luminance (Y) component of Figure 3a. Because of the recovery of the compressed gray values, the number of gray values in Figure 3b is more than that in Figure 2f.

Most images obtained by applying the JNDCA and CPR processes recover most of their lost features, and have better visual enhancement effects. The enhanced images usually have no remaining free spaces. If free spaces remain in an image, the DTE process is applied to enhance the detailed textures of the image.

### 2.3. Detailed Texture Enhancement (DTE)

The third process of VCEA is DTE. The main purpose of DTE is to enhance the detailed textures of an image and make them look clearer. It is usually not easy for people to discern the detailed textures with a few pixels of an image. Thus, the DTE process focuses on those textures for further enhancement.

The DTE process first calculates the gradient value of each pixel, which is the sum of horizontal and vertical gradients. For example, $n_{i,j}$ represents the pixel value of a pixel located at (i,j) in an image with M×N pixels. The gradient value $G_{i,j}$ of $n_{i,j}$ is as follows:

(4) $$G_{i,j}=\left|n_{i,j+1}-n_{i,j-1}\right|+\left|n_{i+1,j}-n_{i-1,j}\right|,\;0\leq i\leq M-1\;and\;0\leq j\leq N-1$$

The DTE process then accumulates the total gradient value and the count of each gray value. It is assumed that *G*(*x*) and *count*(*x*) denote the total gradient value and the count at gray value *x*, respectively. The average gradient value at gray value *x*, *avg*(*x*), is equal to *G*(*x*) divided by *count*(*x*), and is expressed:

(5) avg(x)=G(x)/count(x),  0≤x≤255

Then, the DTE process calculates the mean and the standard deviation gradient of the image. It is assumed that $G_{mean}$ and $G_{\sigma }$ denote the mean and the standard deviation gradient of the image, respectively, and are expressed as follows:

(6) $$G_{mean}=\left(\sum_{i=0}^{M-1}\sum_{j=0}^{N-1}G_{i,j}\right)/\left(M\times N\right)$$

(7) $$G_{\sigma }=\sqrt{\left(\sum_{i=0}^{M-1}\sum_{j=0}^{N-1}{\left(G_{i,j}-G_{mean}\right)}^{2}\right)/\left(M\times N\right)}$$

**Figure 4:**
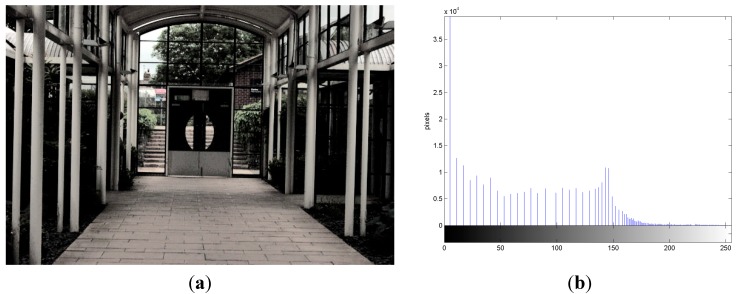
“Indoor View” processed by detailed texture enhancement (DTE). (**a**) DTE image; (**b**) DTE histogram.

Following this, the DTE process determines the candidate gray values to be further enhanced. Here, DTE uses the gradient value as the basis to determine the candidate gray values in order to enhance the detailed textures. This is because a larger gradient value indicates that the relevant pixel is significantly different from adjacent pixels and is much easier to discriminate from them. On the contrary, a small gradient value indicates that the relevant pixel is similar to adjacent pixels and thus is hard to discriminate from them. To render the enhanced effect more obvious, the values of the total number of pixels of the candidate gray values cannot be small. They must be greater than the threshold value, M×N×0.001, where *M* and *N* denote the height and width of an image, respectively. At the same time, the average gradient value of the candidate gray value has to be less than the specific value, which is the absolute value of the difference between $G_{mean}$ and $G_{\sigma }$. Having obtained the qualified candidate gray values, the DTE process sorts them by their average gradient values, and sequentially enhances the candidate gray value with the greater average gradient until all the remaining free spaces are used up. For example, it is assumed that *y* is the first candidate gray value of the CPR histogram to be enhanced. The space between *y* − 1 and *y* is *d* gray levels, and *CPRhist*(*y*) denotes the histogram of the CPR image at gray value *y.* When *d* is greater than *S*(*y*), which is the space adjustment function introduced earlier, *CPRhist*(*y*) is shifted back by *d* − *S*(*y*) gray levels; conversely, when *d* is equal to or less than *S*(*y*)*,* *CPRhist*(*y*) is shifted forward by *S*(*y*) − *d* gray levels. Once all *CPRhist*(*y*) have been sequentially shifted back or forward, the DTE image is obtained. Figure 4a is the image processed by using DTE, and it contains 119 gray values. Through the DTE process, detailed textures such as grass, trees on the left and right side, and the view behind the door in Figure 4a are enhanced. This process also makes the image appear much clearer than the CPR image, indicating that the DTE process can effectively enhance the detailed textures of images. Figure 4b is the histogram of the luminance (Y) component of Figure 4a. It is clear that the dynamic range observed in Figure 4b is wider than that observed in Figure 3b after the DTE process. Therefore, Figure 4a has better image quality. In addition, in this process, all relevant variables are automatically calculated according to the input images, and no parameters need to be tuned manually.

## 3. Experimental Results

**Figure 5:**
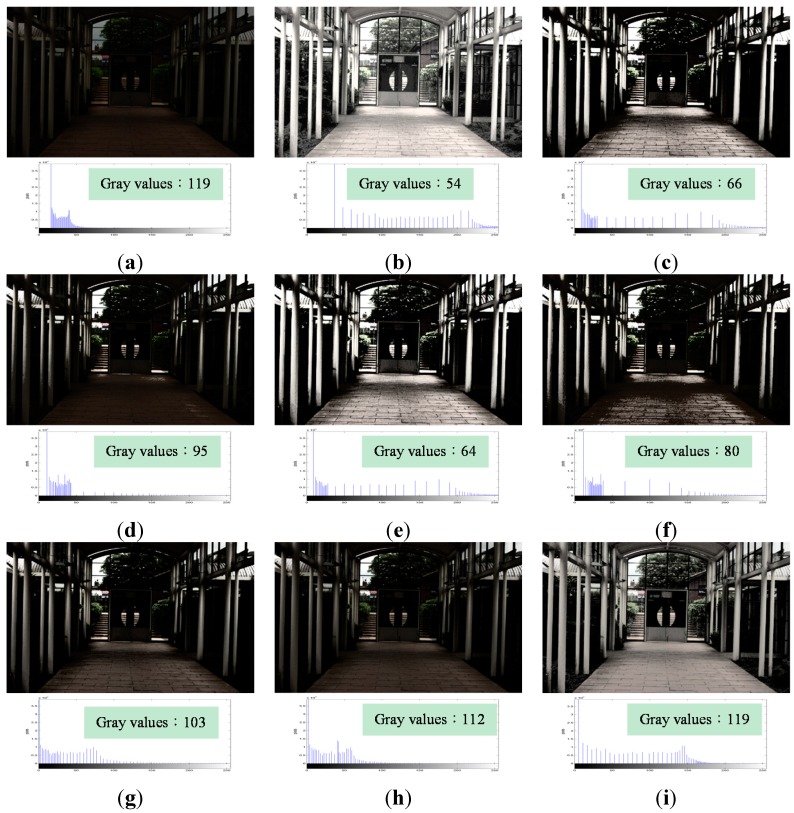
Comparison results for the image “Indoor View” (image size: 640 × 428 pixels). (**a**) Original image; (**b**) Histogram Equalization (HE); (**c**) Bi-histogram equalization (BBHE); (**d**) Recursive mean-separate histogram equalization (RMSHE) (*r* = 2); (**e**) Dualistic sub-image histogram equalization (DSIHE); (**f**) Recursive sub-image histogram equalization (RSIHE) (*r* = 2); (**g**) Bi-histogram equalization with a plateau level (BHEPL); (**h**) Dynamic quadrants histogram equalization plateau limit (DQHEPL); (**i**) Visual contrast enhancement algorithm (VCEA).

**Figure 6:**
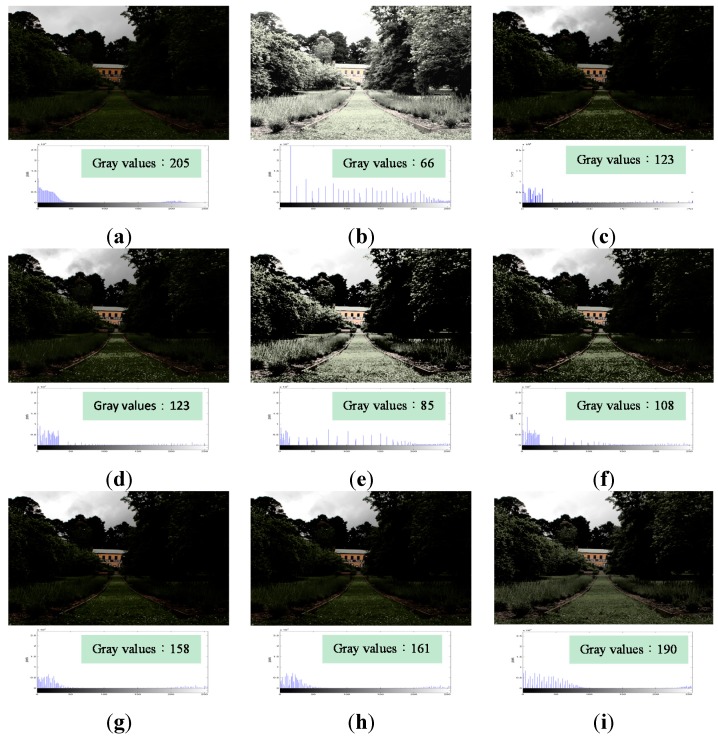
Comparison results for the image “Landscape” [23] (image size: 596 × 397 pixels). (**a**) Original image; (**b**) Histogram Equalization (HE); (**c**) Bi-histogram equalization (BBHE); (**d**) Recursive mean-separate histogram equalization (RMSHE) (*r* = 2); (**e**) Dualistic sub-image histogram equalization (DSIHE); (**f**) Recursive sub-image histogram equalization (RSIHE) (*r* = 2); (**g**) Bi-histogram equalization with a plateau level (BHEPL); (**h**) Dynamic quadrants histogram equalization plateau limit (DQHEPL); (**i**) Visual contrast enhancement algorithm (VCEA).

**Figure 7:**
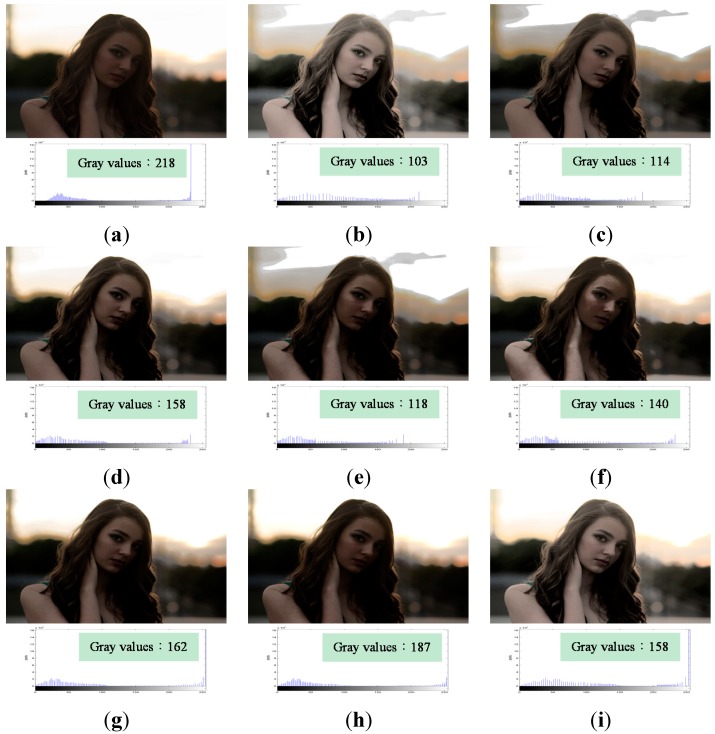
Comparison results for the image “Girl” [24] (image size: 1200 × 800 pixels). (**a**) Original image; (**b**) Histogram Equalization (HE); (**c**) Bi-histogram equalization (BBHE); (**d**) Recursive mean-separate histogram equalization (RMSHE) (*r* = 2); (**e**) Dualistic sub-image histogram equalization (DSIHE); (**f**) Recursive sub-image histogram equalization (RSIHE) (*r* = 2); (**g**) Bi-histogram equalization with a plateau level (BHEPL); (**h**) Dynamic quadrants histogram equalization plateau limit (DQHEPL); (**i**) Visual contrast enhancement algorithm (VCEA).

**Figure 8:**
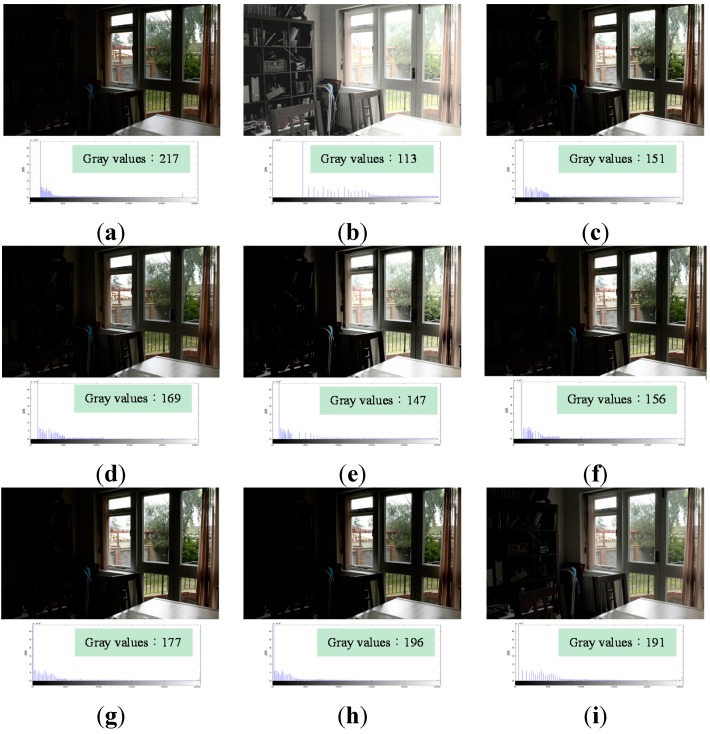
Comparison results for the image “Window View” [25] (image size: 752 × 500 pixels). (**a**) Original image; (**b**) Histogram Equalization (HE); (**c**) Bi-histogram equalization (BBHE); (**d**) Recursive mean-separate histogram equalization (RMSHE) (*r* = 2); (**e**) Dualistic sub-image histogram equalization (DSIHE); (**f**) Recursive sub-image histogram equalization (RSIHE) (*r* = 2); (**g**) Bi-histogram equalization with a plateau level (BHEPL); (**h**) Dynamic quadrants histogram equalization plateau limit (DQHEPL); (**i**) Visual contrast enhancement algorithm (VCEA).

**Figure 9:**
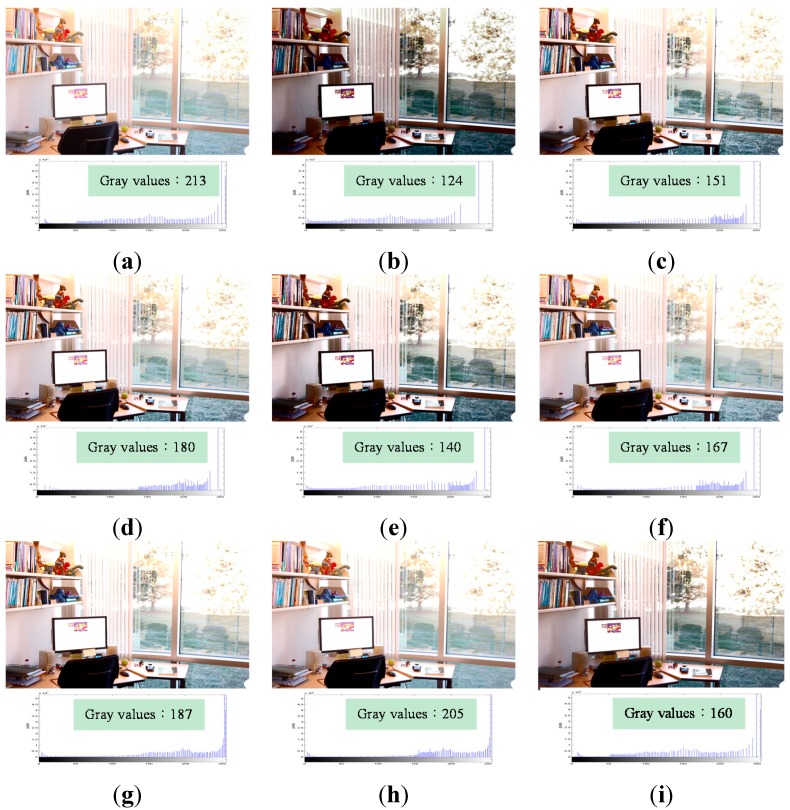
Comparison results for the image “Office” [27] (image size: 903 × 600 pixels). (**a**) Original image; (**b**) Histogram Equalization (HE); (**c**) Bi-histogram equalization (BBHE); (**d**) Recursive mean-separate histogram equalization (RMSHE) (*r* = 2); (**e**) Dualistic sub-image histogram equalization (DSIHE); (**f**) Recursive sub-image histogram equalization (RSIHE) (*r* = 2); (**g**) Bi-histogram equalization with a plateau level (BHEPL); (**h**) Dynamic quadrants histogram equalization plateau limit (DQHEPL); (**i**) Visual contrast enhancement algorithm (VCEA).

Figure 5, Figure 6, Figure 7, Figure 8 and Figure 9 show experimental results for VCEA in comparison with those for HE [1] and other HE-based methods: brightness-preserving bi-histogram equalization (BBHE) [2], recursive mean-separate histogram equalization (RMSHE) [11], equal area dualistic sub-image histogram equalization (DSIHE) [10], recursive sub-image histogram equalization (RSIHE) [14], bi-histogram equalization with a plateau level (BHEPL) [7], and dynamic quadrants histogram equalization plateau limit (DQHEPL) [17].

Figure 5a shows an original image that was underexposed. It contains 119 gray values. Figure 5b shows the image following the processing by using HE. Due to the feature loss problem caused by HE, Figure 5b only contains 54 gray values. This results in the disappearance of the textures of the rain shelter. In addition, the door and rain shelter in the image are over-enhanced, making the colors in the image appear unnatural, particularly the color of the door. Figure 5c,e show the results following the application of BBHE and DSIHE, respectively. They exhibited the same problem of the excessively dark appearance of dark regions and the extremely bright appearance of bright ones. Because of this, many details in the dark and bright regions were not visible. Figure 5d,f were obtained by applying RMSHE and RSIHE, respectively. These had the color distortion problem that made the color of the floor appear very unnatural. Figure 5g,h are the results of processing through BHEPL and DQHEPL, respectively. These appeared too dark, and this rendered invisible some details in the dark regions of the images. However, Figure 5i, the image obtained by applying VCEA, has the same number of gray values as the original image. VCEA not only solves the over enhancement problem caused by HE but also recovers the compressed gray values to make the textures of the rain shelter reappear. It makes Figure 5i show the details in the dark regions most clearly. The image appears more natural, and has higher contrast. In addition, it is suitable for human visual perception.

Figure 6a shows an underexposed image, which contains 205 gray values. Figure 6b shows the image obtained as a result of processing the original image using HE. It contains only 66 gray values and has the feature loss problem. For example, the textures of the house disappear. The image is over-enhanced, and produces the excessive contrast enhancement problem that causes the grass on the road, the leaves on the trees, and the house to become too bright to see. Figure 6c,d, and f show the results of applying BBHE, RMSHE, and RSIHE, respectively to the original image. These exhibited the same problem whereby some regions, like the grass and leaves, appeared unnatural. Figure 6e shows the result of processing the original image using DSIHE, and appears to have the same problem as that encountered in HE processing, *i.e*., some regions, such as the grass and leaves, are too bright to be seen. Figure 6g,h were obtained by applying BHEPL and DQHEPL, respectively. The resulting images are extremely dark, and details such as the grass and leaves cannot be seen clearly as a consequence. However, Figure 6i, which is the result of applying VCEA, contains 190 gray values. Compared to other images, it has the largest number of gray values. The grass, leaves, and house can be seen clearly. The image appears more natural and has higher contrast than that obtained using the other methods. In addition, the obtained image is suitable for human visual perception.

Figure 7a is an underexposed image as well. It contains 218 gray values. Figure 7b–g represent images resulting from the application of HE, BBHE, RMSHE, DSIHE, RSIHE, and BHEPL, respectively. They exhibit the same problem of unpleasant visual artifacts in the background. Figure 7e,f suffer from the color distortion problem, which results in enhanced images appearing unnatural, especially the color of the face. Figure 7h, obtained by applying DQHEPL, yields a better result than the other methods but is a bit dark. Among all the comparison methods, Figure 7g,h) have 162 and 187 gray values, respectively. They have more gray values than Figure 7i, which contains 158 gray values. However, Figure 7i, the image resulting from the application of VCEA, is the clearest and contains no unpleasant visual artifacts in the background. It looks more natural than images obtained by using the other methods.

Figure 8a shows an original underexposed image, which contains 217 gray values. Figure 8b, processed using HE, contains 113 gray values. It is over-enhanced, and produces excessive contrast enhancement, whereby the outdoor view is too bright to be seen. Furthermore, the number of gray values decreases and results in the feature loss problem that the textures of the things on the desk, the grass on the ground, the view, wall, and trees outside the window are difficult to be seen. Figure 8c, the result of applying BBHE, is better than the original image. The objects on the bookshelf in Figure 8c are clearer than in the original image, but are still too dark to see. Figure 8d–h show the results of applying RMSHE, DSIHE, RSIHE, BHEPL, and DQHEPL, respectively. They exhibit the same problem, whereby the objects on the bookshelf are too dark to see. However, Figure 8i, the image obtained by applying VCEA, contains 191 gray values. It has the second largest number of gray values among all the images using other comparison methods. Compared to Figure 8h, which has 196 gray values, Figure 8i shows more clearly the objects on the bookshelf, as well as the outdoor view. In comparison with images obtained by the other methods, this one appears more natural and has a better enhancement effect.

Figure 9a shows an original overexposed image, which contains 213 gray values. Figure 9b shows the image processed using HE. It contains 124 gray values. The back of the chair in Figure 9b is too dark to be seen clearly and some features, such as the textures of the chair back and the paper tray, are lost due to the feature loss problem of HE. Figure 9c,e show the results after the application of BBHE and DSIHE, respectively. The feature loss problem occurs in these images as well because of which the back of the chair is not as clear as the original one. Figure 9d,f–h show the results after the application of RMSHE, RSIHE, BHEPL, and DQHEPL, respectively. Here, the outdoor view and the blinds are too bright to be seen clearly. Figure 9i, which is processed by applying VCEA, contains 160 gray values. Although it has fewer gray values than the ones processed by RMSHE, RSIHE, BHEPL, and DQHEPL, it shows an image, where the blinds and the outdoor view are clearer than those shown in Figure 9a–h. In comparison with images obtained by the other methods, the image processed by VCEA appears more natural and has superior enhancement effects.

In summary, Figure 5, Figure 6, Figure 7, Figure 8 and Figure 9 indicate clearly that VCEA has superior enhancement effects to the other methods that were tested. VCEA not only improves the drawbacks of HE, namely, the excessive contrast enhancement problem and the feature loss problem, but also lends better visual effects and a more natural look to the image. It can also enhance detained textures of images and render them clearer. Compared with HE and other HE-based methods, VCEA produces enhanced images that have superior visual quality and are suitable for human visual perception.

In addition to the above subjective evaluation of the enhancement effect through observation, discrete entropy [26] is used in this study to quantitatively evaluate the effectiveness of the proposed algorithm. It mainly evaluates the capability of the proposed method and other comparison methods for extracting details from images. Discrete entropy *E*(*y*) is defined as:

(8) $$E\left(y\right)=-\sum_{i=0}^{255}p(Y_{i})\times \log_{2}p\left(Y_{i}\right)$$

where $p(Y_{i})$ is the probability of the *i-*th gray level. The higher the entropy value, the more information is extracted from images. The discrete entropy values calculated for different methods are listed in Table 1.

Both subjective and objective assessments are usually used to evaluate the effects of image enhancement. However, researchers often use objective quality assessment, producing results that may not correlate well with human visual perception. Thus, subjective assessment is regarded as the more reliable method for assessing image quality because it measures the most direct response from end users. Objective assessment provides readers quantitative information; however, quantitative information is not enough for people to evaluate the effects of image enhancement. It must be accompanied by subjective assessments. When subjective and objective assessments are not consistent, subjective assessments become more important especially in evaluating the effects of image enhancement.

As seen in Table 1, VCEA shows the highest entropy for Figure 5 and Figure 6, indicating that VCEA extracts considerable information from the original images. Figure 5i has higher contrast and is not over-enhanced. The textures such as the grass on the left and right sides of Figure 5i and the trees behind the door are clearer. Figure 6i also has higher contrast. Textures such as the grass and trees are much clearer. The image is not over-enhanced, either. Therefore, in both objective and subjective assessments, VCEA outperforms the other comparison methods and exhibits a better enhancement effect.

**Table 1:** Calculated discrete entropy values for the compared methods.

Method	Indoor View (Figure 5)	Landscape (Figure 6)	Girl (Figure 7)	Window View (Figure 8)	Office (Figure 9)
HE	5.119617	5.286821	5.968813	5.829778	6.428654
BBHE	5.168818	5.425362	5.993828	5.900343	6.498654
RMSHE	5.103576	5.401507	6.097461	5.927119	6.512488
DSIHE	5.162673	5.374285	6.022877	5.989769	6.473243
RSIHE	5.108531	5.379086	6.054375	5.926646	6.5258
BHEPL	5.224879	5.477687	6.139931	6.038548	6.627142
DQHEPL	5.180584	5.479637	6.186656	6.076967	6.633046
VCEA	5.231328	5.483696	6.070287	6.068298	6.506406

In addition, VCEA has the fourth highest entropy in Figure 7, the second highest entropy in Figure 8, and the fifth highest entropy in Figure 9. Although VCEA cannot extract more details from those images through objective assessment; however, VCEA has better enhancement effects in subjective assessment. For example, in Figure 7i, the face and hair of the girl are much clearer. There is no artifact, such as false contours, shown in Figure 7b,c,e. Figure 7i is more natural and has better enhancement effects than the ones that have higher entropies. In Figure 8i, the entropy is lower than that in Figure 8h. However, the detail textures in the dark area of the image such as the items on the bookshelf can be seen. The enhancement effect of Figure 8i is much better than that of Figure 8h and other comparison methods. In Figure 9, the entropy of Figure 9i is also lower than the ones of Figure 9d,f–h. However, compared to the contrast of these images, the contrast of Figure 9i is higher. The outdoor view and the small image on the screen are much clearer. Figure 9i has better enhancement effects.

In addition to quantitative analyses, in order to demonstrate the superiority of VCEA in subjective assessments, an experiment called “Subjective Image Quality Assessment Test” was designed and conducted by us according to the standard ITU-T P.910 (04/2008)—Subjective video quality assessment methods for multimedia applications. The purpose of the experiment was to provide more subjective assessments for each image from unknown subjects. In this experiment, the absolute category rating (ACR), which is one of the most popular subjective measures in a quality test, was adopted and standardized for images and video in ITU-T P.910. The five-level scale—bad (1), poor (2), fair (3), good (4), and excellent (5)—was used to rate the overall quality of the image. Thirty volunteers without receiving any image processing training on campus were recruited to deliver their assessments. Ten subjects took the “Subjective Image Quality Assessment Test” at a time. They were given the same instructions and 10 s to look at each image. Then, they had to score each image within 10 s. The total scores of all images for different methods are listed in Table 2. As seen in Table 2, VCEA not only shows the highest scores for each image, but also has the highest total score among all the methods. It indicates that the image processed by VCEA has better image quality than the ones obtained by the other methods.

**Table 2:** Calculated scores for each image processed by the compared methods

Method	Indoor View (Figure 5)	Landscape (Figure 6)	Girl (Figure 7)	Window View (Figure 8)	Office (Figure 9)	Sum
HE	116	97	108	125	129	575
BBHE	70	90	93	79	70	402
RMSHE	41	94	71	80	76	362
DSIHE	94	90	54	77	87	402
RSIHE	61	96	38	72	80	347
BHEPL	103	76	89	77	82	427
DQHEPL	106	99	76	84	96	461
VCEA	133	140	132	129	134	668

To sum up, although VCEA extracts fewer details from Figure 7, Figure 8 and Figure 9 compared to those extracted by other methods, the VCEA image is clearer and has higher contrast; the detailed textures are much clearer as well. Through subjective assessments conducted using 30 unknown subjects, it was shown that VCEA has the highest score for each image. This implies that the image processed by VCEA has better image quality than the ones obtained by the other methods. Overall, the subjective and objective analyses indicate that VCEA outperforms other methods and has a better contrast enhancement effect.

## 4. Conclusions

In this paper, a contrast enhancement algorithm called VCEA, which improves image quality in consideration of the requirements of human visual perception, is proposed. VCEA uses the concept of JND to devise a space adjustment function as an adjustable reference to adjust the spaces between two adjacent gray values, which are overstretched by HE, and hence solves the excessive contrast enhancement problem. It is worth noting that VCEA mitigates the feature loss problem, caused by HE or HE-based methods because many gray values are compressed to the same gray value. Further, VCEA aims at representing the detailed textures of an image through further enhancement. Hence, images processed by VCEA have superior visual quality to those obtained using HE and other HE-based methods, and are adequate for human visual perception.
